# Heterotopic Ossification Encountered During a Complex Ventral Hernia Repair: Case Report and Literature Review

**Published:** 2017-09-20

**Authors:** Takintope Akinbiyi, Sanjeev Kaul

**Affiliations:** ^a^Department of General Surgery, Rutgers New Jersey Medical School, Newark, NJ; ^b^Department of General Surgery, Hackensack University Medical Center, Hackensack, NJ

**Keywords:** ectopic/heterotopic ossification, soft-tissue calcification, myositis ossificans traumatica, ventral hernia repair, abdominal wall calcification

## Abstract

**Introduction:** Heterotopic ossification involves the formation of trabecular bone outside of its usual anatomic location. While it is a well-known entity in orthopedic and spinal injury literature, it has also been observed after midline laparotomy and severe burns. **Methods/Case Report:** We present a case of a 69-year-old man who presented for ventral hernia repair after a prolonged postoperative course following colectomy involving an open abdomen with eventual closure with skin grafting. **Results:** Two large calcified objects were encountered during the excision of the skin graft from the small intestine and during the component separation. They had grown into the anterior fascia and rectus muscle and interdigitated between loops of the small bowel. After careful resection of the 2 calcified objects, a ventral hernia repair with a component separation was successfully performed. Pathology was consistent with heterotopic ossification. After 18 months, there was no clinical evidence of recurrence. **Discussion:** Heterotopic ossification is not frequently encountered during ventral hernia repairs, but its presence can complicate repair. Resection is the only option in the context of hernia repair. If recognized preoperatively, waiting up to a year for the bone to mature before excision has been suggested, but there is minimal data to support this. Consultation with a general surgeon is also advised in case the calcified tissue involves the underlying viscera.

Heterotopic ossification (HO) involves the formation of trabecular bone outside of its usual anatomic location and has been described in various forms and under various names since the 18th century.^1^^,^^2^ It is distinct from pathologies such as calcinosis and ectopic calcification because pathologically it comprises structural components similar to bone as opposed to the deposition of calcium in tissue.^3^^,^^4^ Although multiple subtypes have been described, the most relevant to ventral hernia repair is myositis ossificans traumatica.^5^ This condition results in the development of ossified tissue in muscles, tendons, and ligaments^6^ and is frequently observed in the spinal injury and orthopedics literature. Heterotopic ossification of varying degrees of severity and clinical significance has been documented in 5% to 90% of patients following total hip arthroplasty,^6^^,^^7^ in 11% of patients following traumatic brain injury, and in 20% of patients following spinal cord injuries.^8^

While HO is a known sequela of severe neurological injury and arthroplasty, it has also been documented in patients following trauma or severe burns, as well as after laparotomy for abdominal surgery.^4^^,^^5^^,^^9^^-^15 It is estimated that up to 25% of midline wounds develop some amount of HO.^11^ Heterotopic ossification has been observed in all age groups including young children.^16^ The true incidence of HO is unknown but is likely higher due to underreporting. To our knowledge, we present the first case of HO of the ventral abdominal wall and peritoneal cavity that invaded in an almost malignant fashion.

## METHODS/CASE REPORT

**Figure 1:**
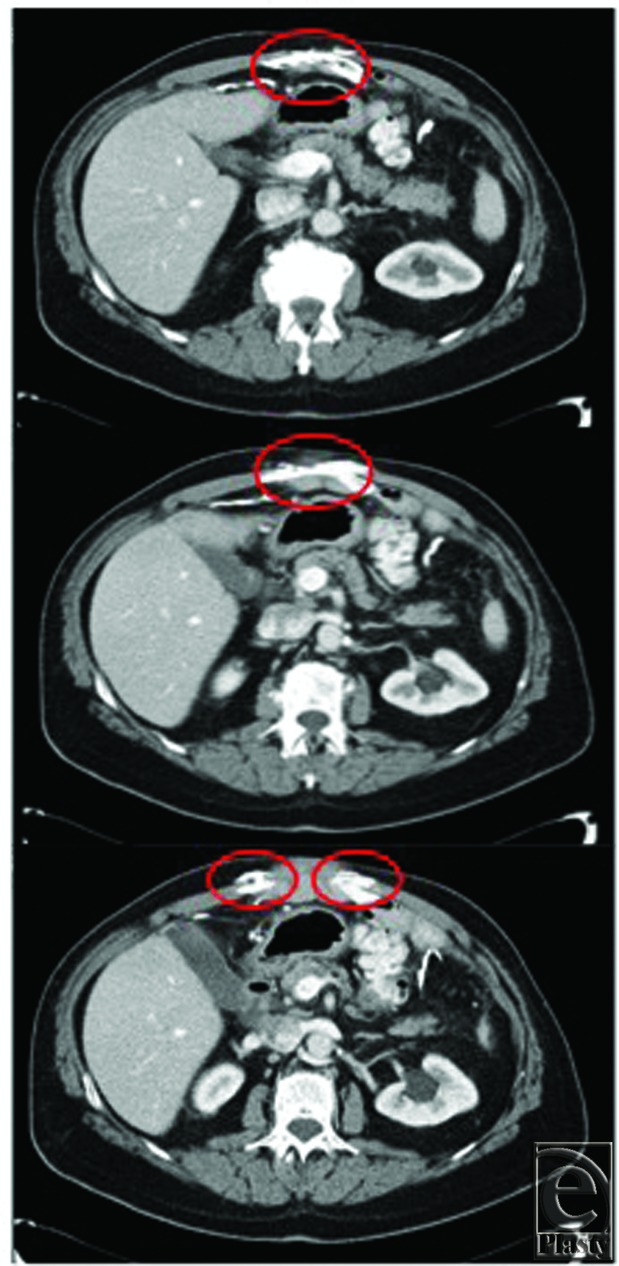
Sequential axial computed tomographic scans of abdominal wall showing cephalad heterotopic ossification invading into rectus muscles and interdigitating between the small bowel.

A 69-year-old obese man with a history of hypertension, hyperlipidemia, benign prostatic hypertrophy, coronary artery disease, and morbid obesity was diagnosed in 2010 with rectal cancer. He underwent a robot-assisted abdominoperineal resection complicated by an intraoperative injury to the right ureter requiring conversion to laparotomy. His postoperative course was complicated by abdominal compartment syndrome, requiring emergent decompressive laparotomy, and by a severe surgical site infection of his laparotomy incision. The incision eventually broke down completely, with dehiscence of the fascia resulting in an open abdomen. During an extended intensive care unit course, his abdomen was temporarily closed with a negative therapy vacuum dressing. When stable, he underwent split-thickness skin grafting directly on his viscera for coverage with continued negative pressure vacuum dressing until adequate take was achieved. After discharge and complete recovery, he developed complete loss of domain and a large ventral hernia that was symptomatic. Four years later, he presented to our clinic requesting elective repair of his ventral hernia. An otherwise unremarkable preoperative workup was notable for the computed tomographic (CT) scan demonstrating the lateralized rectus muscle with a large fascial defect and calcification within the rectus sheath, muscle, and interdigitating within the anterior small bowel (Fig 1).

## RESULTS

**Figure 2:**
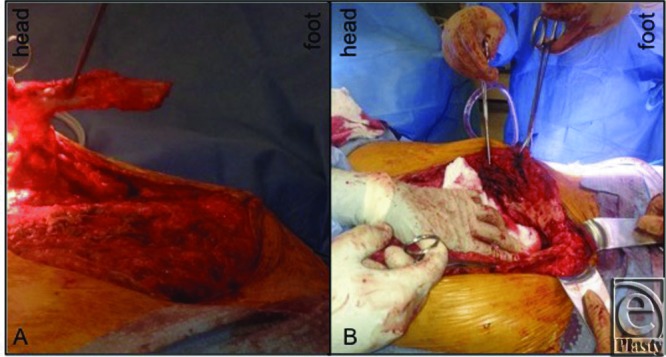
Intraoperative picture showing the cephalad (a) and caudal (b) heterotopic ossified tissue within the abdominal wall.

**Figure 3:**
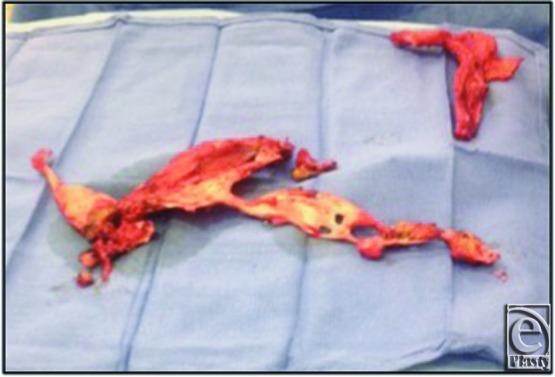
Specimens after resection. The specimen on the left was caudal, and the right specimen was cephalad in the ventral abdominal wall.

His planned surgery involved removal of the prior split-thickness skin graft and a component separation of his abdominal wall, now possible due to planned extensive weight loss, to fix the ventral hernia. In addition, the calcified objects would be removed during the dissection. During the procedure, the skin graft was found to be densely adherent to the underlying small bowel. Several enterotomies were created along multiple segments of the small bowel during skin graft excision. The enterotomies were treated by resection and primary anastomosis. During the dissection, 2 areas of calcified tissue were encountered, the extent of which had not been fully appreciated on preoperative imaging. The cephalad calcified object was in the shape of a small antler just below the xiphoid. The second object was caudal and much larger in size. It had a horseshoe-like configuration that appeared to originate just above the pubic symphysis and its 2 horns spread laterally and cephalad (Figs 2 and 3).

The 2 ossified objects were sharply dissected free, minimizing further injury to the small bowel. This required longitudinal incision of the left anterior rectus fascia and muscle. The cephalad and caudal structures were divided from the xiphoid and pubic symphysis, respectively, with orthopedic cutters. The remainder of the case was uneventful with bilateral component separation and local advancement flaps of the anterior rectus fascia. Final pathology documented bone with cortical reactive sclerosis consistent with HO. Postoperatively, the patient did well after an initial ileus and was discharged home without issue. At 18 months, he had no clinical evidence of recurrence.

## DISCUSSION

Heterotopic ossification involves the formation of extraskeletal trabecular bone between muscle planes and is a known sequela of orthopedic surgery,^6^^,^^7^^,^^17^ severe neurological injury,^7^^,^^8^^,^^18^^,^^19^ traumatic injury,^19^^-^22 and less frequently of surgical intervention^4^^,^^5^^,^^9^^-^15 and burns.^23^^-^25 It can be distinguished from truly malignant neoplasms such as osteosarcoma or osteochondroma on the basis of its radiographic and histological properties. Histologically, HO initially appears as a focus of inflammatory infiltration and progresses to areas of osteoid or cartilaginous tissue formation. On occasion, mature HO can resemble lamellar bone.^1^ Radiographically, it can appear as a radiopaque rim surrounding a radiolucent center, an appearance dissimilar to that of malignant lesions, although there is a high degree of overlap.^2^^,^^26^^,^^27^

Some reports suggest that patients may have a genetic predisposition to HO with a higher expression of the HLA-B27, HLA-B18, and HLA-DRW7antigens in neurogenic HO.^1^^,^^8^ In patients with neurogenic impairment, traumatic brain injury or spinal injury, superimposed trauma has also been found to increase the rate of HO both in animal models and in clinical observational studies.^6^^,^^8^ Other observed risk factors include male sex, young age (20-30 years), completeness of any associated spinal lesions, and prolonged coma.^8^^,^^10^

While the etiology of HO is unknown, it is assumed to be multifactorial. It is speculated that the calcified tissue found in HO may be the result of the transformation of mesenchymal cells in the connective tissue septa of muscle into osteogenic cells.^9^^,^^28^ The role of mesenchymal cells is supported by the finding that bone morphogenetic proteins (BMP), which are known to induce mesenchymal stem cells toward ostoblastic differentiation, have been speculated to exist in supraphysiologic concentrations around areas of trauma or surgical incisions.^2^^,^^24^^,^^29^ In addition, a mutation in a BMP-type I receptor has been associated with inherited and sporadic fibrodysplasia ossificans progressiva, a rare condition predisposing to HO.^30^ The role of inflammation has been suggested by many authors and studied in experimental models and has been found to play a strong role.^24^ Baird and Kang^6^ proposed that HO could also be mediated by systemic factors, local inflammation, cell death, upregulation of mineralization growth factors, or ischemia-induced free radical production leading to tissue injury. The development of HO may also partially be due to venous stasis or damage to bone and its surrounding connective tissue.^9^

While HO has been anecdotally observed in abdominal incisions, little is known about its true incidence or characteristics.^12^ Kim et al^11^ retrospectively looked at CT scans from 152 postoperative patients with abdominal incisions, approximately 50% involving upper midline incisions, and found radiographic evidence of HO in 25%. The mean time to follow-up was 378 days.^11^ Jacobs et al^12^ retrospectively reviewed 11 patients with HO and found that they all had undergone upper midline incisions and developed HO between the anterior abdominal fascia and the peritoneum, originating in close proximity to the xiphoid. Mean follow-up was 6.7 months.^12^ Another retrospective review over 9 years found 7 cases of HO, all between the fascia and the peritoneum in upper midline incisions.^14^ In a review of reported cases of HO published between 1920 and 1998, Gaffey and Winston^5^ found 130 cases of HO described in surgical incisions. Of the cases with available clinical information, 72% had palpable lesions of which 62% were located in upper midline incisions.^5^ While the majority of suspected lesions in the aforementioned case series were not sampled for pathology, a diagnosis of HO over calcinosis was made by the authors on the basis of radiographic appearance or clinical examinations.

### Prophylaxis and treatment

Multiple groups have studied prophylaxis of HO in high-risk populations. Nonsteroidal anti-inflammatory drugs have been shown to reduce HO formation by inhibiting prostaglandin-mediated bone remodeling, especially PGE-2.^6^^,^^17^^,^^31^ Free radical scavengers, allopurinol and *N*-acetylcysteine, have been shown in experimental models to reduce HO formation.^32^ Bisphosphonates are known to limit the mineralization of organic osteoid and have also been shown to inhibit or limit the formation of HO in the spinal cord injury population.^18^^,^^33^ Radiation therapy is another treatment modality known to inhibit HO formation. Studies in the orthopedic literature have shown that radiation therapy can reduce HO formation and has been used as prophylaxis against its formation.^34^^,^^35^ However, radiation therapy has been associated with posttreatment complications including seroma formation.^36^ Finally, warfarin has been studied as a prophylactic agent, as osteocalcin, an osteoblast-specific noncollagenous protein important in bone development, is vitamin K dependent.^37^

While prophylactic measures have shown benefit in the neurogenic injury and orthopedic literature, abdominal incisions are too common to justify the expense of routine HO prophylaxis. Furthermore, exposing patients to the risks of the side effects of abdominal radiation or systemic anticoagulation is not appropriate. In ventral hernia repair with mesh, seroma formation after radiation is also undesirable. Therefore, surgical resection currently appears to represent the sole viable treatment option.

There is limited information available to guide timing and technique of resection. Some authors have suggested that only mature, symptomatic HO be excised,^13^^,^^15^^,^^33^ as immature lesions may have a higher rate of recurrence.^38^^,^^39^ Koolen et al^10^ attempted a systematic review of the literature pertaining to the treatment of HO in abdominal incisions but were unable to find any randomized control trials, only case reports. This dearth of knowledge has contributed to the uncertainty in managing symptomatic HO in burned patients and ventral abdominal incisions. Some authors have advocated a 12- to 18-month delay before surgical excision to reduce the likelihood of recurrence,^40^^,^^41^ but this is in regard to HO in neurogenic patients and may not be necessary in HO identified in burn injuries and abdominal incisions.

Objective evidence supporting delaying resection is lacking.^42^^-^45 Trending the levels of creatine phosphokinase^2^ and serum alkaline phosphatase or using bone scans as markers of maturation has been suggested, but the accuracy and clinical utility of these are controversial.^46^ In a study of 37 joints, 25 had postoperative radiographic studies available and 14 of these (55%) had evidence of recurrence. However, neither the extent of recurrences nor the follow-up duration was fully documented.^41^ Conversely, Tsionos et al^43^ evaluated 28 consecutive patients with burn injury involving 35 elbows and hypothesized that in this patient population, early excision would be beneficial. They observed only 4 recurrences (radiographic and/or clinical) after resection, with a mean delay of 12 months from formation. No recurrences were observed in HO originating away from bone, which may be more applicable to HO in abdominal incisions. In addition, another study of 9 elbow joints postoperative resection of HO found no recurrences over a follow-up period of 10 months to 8.7 years.^40^ Therefore, there is some evidence that early excision is appropriate.

### Conclusion

We reported a case of a 69-year-old man who developed HO after a prolonged open abdomen, eventually closed by split-thickness skin grafting. Two large collections of heterotrophic ossified tissue, invading in an almost malignant fashion, were resected during a subsequent ventral hernia repair. To our knowledge, we present the first description of HO that invaded the abdominal wall and intraperitoneal viscera in an almost malignant fashion.

Heterotopic ossification has historically been reported in the orthopedic and spinal injury literature. While not as common, it has also been reported in the trauma and burn literature as well as after midline laparotomy. Studies documenting its radiographic presence in 25% of patients following abdominal surgery suggest that its real incidence is underreported.^11^ Although potential mechanisms have been proposed, the etiology is still unclear and likely multifactorial. However, inflammation has been found anecdotally and experimentally to play a major role.^24^ While the location of formation is varied, the majority of cases appear to form as a sequela of upper midline incisions. The decision to offer surgical treatment is usually based on symptom burden, although our case required excision for completion of the ventral hernia repair. Currently, there is little information on the recurrence rate of HO. Some authors have advocated delaying definitive surgical treatment to decrease the risk of early recurrence; however, there is evidence that early excision is appropriate.
